# Supplementary material for: A Likelihood Approach for Real-Time Calibration of Stochastic Compartmental Epidemic Models
Source: PLoS Comput Biol. 2017 Jan 17;13(1):e1005257. doi: 10.1371/journal.pcbi.1005257 (PMC5240920; doi:10.1371/journal.pcbi.1005257)
Supplement: S1 File — (TAR.GZ) [file pcbi.1005257.s014.tar.gz › HSPH_Online-SI-Revision/Additions/MSS11/n100-extreme-SEITR/n100-extreme-SEITR_NEW2_table-4.pdf]

1	newly 3 weeks spec	13598.	9272.84	{8805., 9905.}	31.8073	0.166722	newly 3 weeks cumul	31455.	24692.6	{23316., 26275.}	21.4985	0.10563	newly 5 weeks spec	12085.	5611.95	{5312., 5860.}	53.5627	0.333463	newly 5 weeks cumul	57301.	38139.2	{36568., 39846.}	33.4407	0.177055	newly 55 weeks cumul	84027.	46723.3	{45623., 48075.}	44.3948	0.254987
2	newly 3 weeks spec	17235.	17207.9	{16446., 18068.}	3.02785	0.0131158	newly 3 weeks cumul	29177.	29653.2	{28051., 31358.}	3.68122	0.0157089	newly 5 weeks spec	17453.	15679.	{14695., 16226.}	10.1644	0.0470296	newly 5 weeks cumul	67910.	65026.2	{63795., 66241.}	4.31225	0.0191897	newly 62 weeks cumul	97717.	90154.6	{82548., 92761.}	7.73905	0.0355113
3	newly 3 weeks spec	14424.	14617.	{11452., 17254.}	12.7651	0.0563725	newly 3 weeks cumul	24222.	25323.8	{19382., 30277.}	15.4561	0.0659399	newly 5 weeks spec	18266.	11523.3	{6976., 15390.}	36.9142	0.219203	newly 5 weeks cumul	61841.	53055.9	{43523., 59110.}	14.4729	0.070599	newly 64 weeks cumul	97783.	65205.3	{49855., 77996.}	33.3164	0.181052
4	newly 3 weeks spec	13822.	11706.7	{10271., 13416.}	15.5765	0.0753278	newly 3 weeks cumul	25848.	23566.4	{20743., 26584.}	10.4232	0.0485979	newly 5 weeks spec	16022.	10383.7	{9026., 12154.}	35.1913	0.191812	newly 5 weeks cumul	59339.	46673.5	{42017., 53232.}	21.3443	0.106049	newly 62 weeks cumul	94062.	62732.3	{57216., 71128.}	33.3075	0.178703
5	newly 3 weeks spec	22578.	16635.	{14824., 18956.}	26.3223	0.135751	newly 3 weeks cumul	36477.	27386.7	{23844., 31370.}	24.9207	0.128449	newly 5 weeks spec	16920.	12895.4	{11009., 14930.}	23.786	0.123682	newly 5 weeks cumul	77798.	59138.7	{53105., 64808.}	23.9843	0.121128	newly 64 weeks cumul	97923.	70982.4	{65457., 77301.}	27.512	0.142391
6	newly 3 weeks spec	20143.	16630.1	{14714., 18794.}	17.44	0.0879144	newly 3 weeks cumul	34123.	30957.2	{25943., 36104.}	11.9718	0.06113	newly 5 weeks spec	17405.	8972.34	{3934., 12273.}	48.4496	0.322701	newly 5 weeks cumul	74866.	54922.8	{45352., 62341.}	26.6386	0.138145	newly 63 weeks cumul	93450.	62754.8	{48464., 74606.}	32.8467	0.179295
7	newly 3 weeks spec	12108.	9050.14	{8597., 9478.}	25.2549	0.126804	newly 3 weeks cumul	28975.	24775.1	{23419., 25978.}	14.495	0.068385	newly 5 weeks spec	11332.	5374.3	{4993., 5711.}	52.5741	0.325915	newly 5 weeks cumul	52923.	37674.3	{36121., 38948.}	28.8129	0.147946	newly 55 weeks cumul	88800.	46072.5	{44626., 46543.}	48.1165	0.285715
8	newly 3 weeks spec	16980.	16329.9	{14952., 17122.}	4.56508	0.0207055	newly 3 weeks cumul	32632.	32050.8	{30296., 34335.}	4.6782	0.0203909	newly 5 weeks spec	14766.	12870.1	{4157., 14561.}	12.8572	0.0967546	newly 5 weeks cumul	65812.	61456.2	{47157., 64518.}	6.62299	0.0319136	newly 62 weeks cumul	97109.	83807.2	{48600., 90272.}	13.6978	0.0716524
9	newly 3 weeks spec	23304.	16457.5	{14356., 18059.}	29.4134	0.153813	newly 3 weeks cumul	35304.	24749.1	{21319., 27682.}	30.044	0.158062	newly 5 weeks spec	17411.	18341.8	{16863., 19414.}	8.02142	0.0341434	newly 5 weeks cumul	78660.	65532.1	{62466., 68040.}	16.6894	0.0798087	newly 64 weeks cumul	98833.	90261.6	{88285., 91941.}	8.67262	0.0400223
10	newly 3 weeks spec	12030.	11458.3	{11032., 11865.}	4.86218	0.0217925	newly 3 weeks cumul	25011.	23668.5	{22607., 24720.}	5.55388	0.024974	newly 5 weeks spec	13614.	12271.	{10739., 12845.}	9.86462	0.046032	newly 5 weeks cumul	52825.	49123.	{46677., 50629.}	7.00806	0.0317323	newly 60 weeks cumul	95503.	80911.2	{67012., 86525.}	15.2789	0.073903
11	newly 3 weeks spec	13896.	12352.4	{10507., 14041.}	12.2376	0.0584891	newly 3 weeks cumul	26326.	23782.3	{20183., 26811.}	10.8902	0.0517011	newly 5 weeks spec	14675.	11978.3	{10266., 12992.}	18.3759	0.0899172	newly 5 weeks cumul	57151.	49900.6	{42530., 54081.}	12.783	0.0611138	newly 59 weeks cumul	97210.	69069.3	{57835., 72374.}	28.9483	0.150045
12	newly 3 weeks spec	16108.	16213.1	{15548., 16675.}	2.41209	0.0104864	newly 3 weeks cumul	33166.	34311.5	{32445., 35575.}	4.45284	0.0189118	newly 5 weeks spec	14159.	11596.3	{10688., 12348.}	18.0992	0.087355	newly 5 weeks cumul	64453.	61448.2	{59797., 62967.}	4.66203	0.020817	newly 60 weeks cumul	95054.	79350.4	{75160., 82003.}	16.5207	0.0787738
13	newly 3 weeks spec	15592.	15526.4	{14386., 16453.}	3.86974	0.0169632	newly 3 weeks cumul	30732.	30418.1	{28091., 32421.}	4.25566	0.018765	newly 5 weeks spec	14704.	13242.6	{12114., 14158.}	9.93886	0.0476801	newly 5 weeks cumul	62511.	60108.9	{57777., 62936.}	4.20182	0.0188844	newly 61 weeks cumul	96828.	83495.6	{76401., 91174.}	13.7691	0.0658306
14	newly 3 weeks spec	10081.	8582.44	{8300., 8985.}	14.8652	0.0709049	newly 3 weeks cumul	23444.	20671.5	{19854., 21447.}	11.8259	0.0550963	newly 5 weeks spec	11642.	8135.86	{8038., 8416.}	30.1163	0.15844	newly 5 weeks cumul	46832.	37721.8	{36919., 38995.}	19.4529	0.0948696	newly 54 weeks cumul	89533.	60052.	{60091., 61171.}	32.9275	0.175253
15	newly 3 weeks spec	14236.	10299.7	{9542., 11067.}	27.65	0.141403	newly 3 weeks cumul	30841.	24660.3	{22589., 26462.}	20.0406	0.097998	newly 5 weeks spec	13230.	7036.36	{6522., 7425.}	46.8151	0.276048	newly 5 weeks cumul	59486.	41172.8	{38779., 42662.}	30.7857	0.160452	newly 55 weeks cumul	91749.	51808.1	{50250., 51911.}	43.5328	0.249129
16	newly 3 weeks spec	11254.	8668.07	{6043., 10033.}	22.9779	0.122532	newly 3 weeks cumul	24092.	20455.7	{15881., 22639.}	15.1457	0.076212	newly 5 weeks spec	12953.	7989.13	{3648., 10987.}	38.3222	0.247178	newly 5 weeks cumul	50447.	37413.6	{24904., 44421.}	25.8358	0.14102	newly 58 weeks cumul	87287.	60057.	{31095., 81593.}	31.1959	0.195477
17	newly 3 weeks spec	15615.	15015.8	{12323., 17190.}	8.75485	0.0404716	newly 3 weeks cumul	24517.	24337.7	{19411., 28833.}	10.6496	0.0477454	newly 5 weeks spec	20574.	15019.1	{11669., 17165.}	26.9994	0.143694	newly 5 weeks cumul	68260.	58386.6	{52468., 62914.}	14.4644	0.0690455	newly 64 weeks cumul	92364.	76302.7	{65851., 83187.}	17.4031	0.0857998
18	newly 3 weeks spec	19007.	14373.5	{13500., 15293.}	24.378	0.122066	newly 3 weeks cumul	31170.	23115.7	{21577., 24771.}	25.8398	0.130688	newly 5 weeks spec	17189.	17293.	{16980., 17909.}	3.75868	0.0169929	newly 5 weeks cumul	70235.	59799.3	{58287., 61730.}	14.8583	0.0701084	newly 63 weeks cumul	98793.	90742.9	{83363., 93158.}	8.14845	0.0381882
19	newly 3 weeks spec	12889.	10671.	{8947., 11884.}	17.2086	0.0841677	newly 3 weeks cumul	24953.	21354.6	{18154., 23628.}	14.4567	0.0698337	newly 5 weeks spec	14542.	10208.1	{8923., 11767.}	29.8024	0.156297	newly 5 weeks cumul	54787.	43586.1	{38095., 48540.}	20.4444	0.100935	newly 58 weeks cumul	96606.	60166.8	{53832., 68217.}	37.7194	0.207578
20	newly 3 weeks spec	19368.	17715.4	{17072., 18398.}	8.53284	0.0389801	newly 3 weeks cumul	33454.	31410.9	{29891., 33048.}	6.1708	0.0281063	newly 5 weeks spec	16864.	15270.5	{14762., 15734.}	9.44889	0.0432945	newly 5 weeks cumul	72041.	66105.5	{65046., 67581.}	8.23911	0.0374353	newly 63 weeks cumul	97397.	91139.2	{84513., 92571.}	6.42503	0.0291527
21	newly 3 weeks spec	19672.	21285.9	{18848., 24255.}	11.6692	0.0477456	newly 3 weeks cumul	30439.	34452.2	{29991., 40258.}	15.3226	0.0607803	newly 5 weeks spec	18543.	15111.3	{13281., 16884.}	18.5066	0.0935651	newly 5 weeks cumul	73114.	71913.4	{69272., 76115.}	4.42535	0.0205848	newly 64 weeks cumul	98246.	89311.4	{88206., 93113.}	9.09413	0.0434254
22	newly 3 weeks spec	14910.	13258.2	{12790., 13642.}	11.0783	0.0511816	newly 3 weeks cumul	31688.	29195.7	{28116., 30180.}	7.86512	0.0357184	newly 5 weeks spec	14003.	10737.7	{9212., 12090.}	23.3187	0.119667	newly 5 weeks cumul	61831.	53203.6	{50389., 55321.}	13.9531	0.0657298	newly 60 weeks cumul	92206.	73766.	{65548., 80337.}	19.9987	0.0999645
23	newly 3 weeks spec	16317.	12354.2	{11458., 13297.}	24.2866	0.121749	newly 3 weeks cumul	34910.	28369.1	{26350., 30219.}	18.7365	0.0908907	newly 5 weeks spec	13260.	8211.38	{7142., 9521.}	38.0741	0.211489	newly 5 weeks cumul	65072.	47879.1	{44598., 51911.}	26.4214	0.134243	newly 61 weeks cumul	83725.	59425.9	{54864., 65905.}	29.0226	0.150708
24	newly 3 weeks spec	16757.	17444.9	{14483., 19202.}	9.68604	0.040976	newly 3 weeks cumul	25641.	27096.4	{22927., 30676.}	10.8384	0.0451301	newly 5 weeks spec	20012.	16784.5	{14618., 18342.}	16.1281	0.080319	newly 5 weeks cumul	69280.	65779.6	{57933., 69812.}	5.98679	0.0281421	newly 63 weeks cumul	98228.	86814.4	{77110., 91792.}	11.6195	0.0565881
25	newly 3 weeks spec	12372.	10884.3	{10417., 11461.}	12.025	0.0559556	newly 3 weeks cumul	23919.	22076.8	{21044., 23526.}	7.90531	0.0360827	newly 5 weeks spec	15457.	12737.4	{12574., 12966.}	17.5949	0.0842106	newly 5 weeks cumul	55191.	47856.5	{46609., 49610.}	13.2893	0.06206	newly 59 weeks cumul	91517.	85482.8	{85818., 86330.}	6.59358	0.0299922
26	newly 3 weeks spec	12283.	9175.47	{8649., 9678.}	25.2994	0.12731	newly 3 weeks cumul	28429.	24180.5	{23043., 25570.}	14.9442	0.0707504	newly 5 weeks spec	11730.	5808.3	{5300., 5995.}	50.4834	0.308085	newly 5 weeks cumul	53475.	37900.7	{36345., 39167.}	29.1245	0.1501	newly 57 weeks cumul	80102.	47269.	{45301., 47634.}	40.989	0.230576
27	newly 3 weeks spec	12409.	11310.3	{10987., 11558.}	8.85446	0.0403494	newly 3 weeks cumul	28757.	27297.6	{26620., 27949.}	5.0748	0.0226932	newly 5 weeks spec	11908.	9593.95	{9284., 10218.}	19.4327	0.0940813	newly 5 weeks cumul	53686.	48135.9	{47275., 48945.}	10.338	0.0474332	newly 57 weeks cumul	90968.	70967.9	{69588., 77740.}	21.9859	0.108124
28	newly 3 weeks spec	16959.	13803.1	{10603., 16069.}	18.6506	0.100588	newly 3 weeks cumul	28083.	25909.2	{16938., 33508.}	20.3121	0.106524	newly 5 weeks spec	18063.	7027.74	{3975., 11082.}	61.0932	0.446582	newly 5 weeks cumul	68202.	45022.4	{41033., 48065.}	33.9867	0.181943	newly 64 weeks cumul	88415.	50467.1	{48072., 52626.}	43.0173	0.24558
29	newly 3 weeks spec	12914.	11925.	{11565., 12339.}	7.65828	0.0347963	newly 3 weeks cumul	24569.	22971.9	{22094., 23792.}	6.51129	0.0294735	newly 5 weeks spec	15388.	13600.5	{13226., 14042.}	11.616	0.0539819	newly 5 weeks cumul	56007.	51068.8	{49721., 52141.}	8.81715	0.0401998	newly 61 weeks cumul	96532.	82087.5	{79930., 88795.}	14.9634	0.0713226
30	newly 3 weeks spec	13125.	16960.5	{15224., 18403.}	29.5506	0.11108	newly 3 weeks cumul	20142.	26122.7	{23046., 28686.}	30.2798	0.113327	newly 5 weeks spec	19690.	17418.4	{15542., 18499.}	11.537	0.0555279	newly 5 weeks cumul	60177.	65545.6	{59882., 68559.}	10.2117	0.0422956	newly 64 weeks cumul	98710.	88255.3	{77319., 91801.}	10.5914	0.0503821
31	newly 3 weeks spec	10370.	4971.81	{4260., 5177.}	52.0558	0.326917	newly 3 weeks cumul	26082.	16794.7	{14508., 18551.}	35.608	0.194506	newly 5 weeks spec	9931.	2530.43	{1901., 2437.}	74.5199	0.632122	newly 5 weeks cumul	46868.	22998.4	{19604., 24204.}	50.9294	0.316165	newly 54 weeks cumul	76589.	27816.4	{23312., 26774.}	63.6809	0.460622
32	newly 3 weeks spec	12614.	12211.	{11990., 12413.}	3.22451	0.014273	newly 3 weeks cumul	26998.	26873.8	{26405., 27303.}	1.19216	0.00519703	newly 5 weeks spec	13001.	11915.2	{11829., 12094.}	8.3519	0.0380642	newly 5 weeks cumul	54036.	51925.7	{51410., 52498.}	3.90543	0.0173215	newly 60 weeks cumul	93822.	86254.1	{86459., 86835.}	8.06621	0.0368217
33	newly 3 weeks spec	20264.	16481.9	{13420., 18685.}	18.6639	0.0931782	newly 3 weeks cumul	37971.	32806.1	{27324., 37544.}	13.884	0.0679287	newly 5 weeks spec	15166.	8405.54	{6144., 11181.}	44.5764	0.274636	newly 5 weeks cumul	73450.	55324.1	{45178., 63942.}	24.6779	0.126551	newly 64 weeks cumul	96763.	62868.3	{51151., 73916.}	35.0286	0.192363
34	newly 3 weeks spec	15602.	12165.9	{11381., 13332.}	22.0237	0.108831	newly 3 weeks cumul	34185.	29404.3	{27263., 31490.}	13.9848	0.0661025	newly 5 weeks spec	13645.	7256.43	{6474., 8864.}	46.8199	0.27723	newly 5 weeks cumul	63916.	47169.3	{44403., 52380.}	26.2011	0.132706	newly 60 weeks cumul	89965.	56832.1	{53942., 65248.}	36.8286	0.200703
35	newly 3 weeks spec	18683.	16712.3	{15145., 18320.}	10.5944	0.0499532	newly 3 weeks cumul	29482.	27261.3	{24304., 30137.}	8.7049	0.0408536	newly 5 weeks spec	19217.	15218.2	{13182., 16649.}	20.8087	0.103452	newly 5 weeks cumul	73185.	62643.3	{57833., 66448.}	14.4042	0.0680694	newly 64 weeks cumul	94833.	80277.9	{74436., 87840.}	15.3481	0.0737103
36	newly 3 weeks spec	11929.	12287.2	{11335., 13168.}	5.87124	0.0247297	newly 3 weeks cumul	21930.	22512.3	{20306., 24076.}	5.85385	0.0247292	newly 5 weeks spec	15024.	14191.5	{13091., 14843.}	5.54746	0.0253104	newly 5 weeks cumul	52068.	52104.9	{49219., 54980.}	3.55235	0.0154923	newly 60 weeks cumul	97587.	79789.7	{71828., 84348.}	18.2374	0.0883429
37	newly 3 weeks spec	15965.	16109.7	{13875., 18151.}	9.11882	0.0398262	newly 3 weeks cumul	25084.	25576.9	{21041., 29210.}	10.7732	0.0467759	newly 5 weeks spec	18951.	15080.6	{13751., 17028.}	20.4231	0.10091	newly 5 weeks cumul	65780.	60856.1	{57104., 64195.}	7.48545	0.0343241	newly 61 weeks cumul	98650.	76198.5	{73341., 77736.}	22.7587	0.112595
38	newly 3 weeks spec	14686.	11559.1	{11326., 11763.}	21.2919	0.104044	newly 3 weeks cumul	31691.	26253.6	{25775., 26695.}	17.1575	0.0818044	newly 5 weeks spec	13535.	11219.8	{10943., 11430.}	17.1052	0.0815356	newly 5 weeks cumul	60924.	49783.2	{49319., 50301.}	18.2865	0.087721	newly 58 weeks cumul	87770.	82848.7	{77273., 84186.}	5.607	0.0253086
39	newly 3 weeks spec	14352.	11710.3	{10665., 12627.}	18.4067	0.0893167	newly 3 weeks cumul	28679.	24776.1	{22611., 26602.}	13.6088	0.0644289	newly 5 weeks spec	15042.	10062.6	{8235., 10902.}	33.1032	0.177783	newly 5 weeks cumul	60279.	47123.7	{42618., 50364.}	21.8241	0.107893	newly 60 weeks cumul	93593.	64414.	{53680., 67744.}	31.1765	0.16472
40	newly 3 weeks spec	14498.	12656.8	{11733., 13334.}	12.6998	0.0595154	newly 3 weeks cumul	31327.	30453.9	{26179., 33845.}	7.25113	0.0325706	newly 5 weeks spec	13194.	5847.27	{4206., 7457.}	55.6824	0.362703	newly 5 weeks cumul	59514.	46094.1	{44396., 47812.}	22.5492	0.111265	newly 61 weeks cumul	94658.	51940.	{48930., 55848.}	45.1287	0.2613
41	newly 3 weeks spec	13435.	10304.5	{8966., 11456.}	23.4767	0.118787	newly 3 weeks cumul	19619.	15459.6	{13076., 17378.}	21.6657	0.108576	newly 5 weeks spec	23349.	18871.1	{18335., 19382.}	19.1783	0.092811	newly 5 weeks cumul	66595.	51941.	{48457., 55154.}	22.0047	0.108559	newly 64 weeks cumul	95882.	90081.2	{80294., 93277.}	6.04996	0.0280838
42	newly 3 weeks spec	10315.	8459.33	{5188., 9810.}	17.99	0.0997374	newly 3 weeks cumul	25161.	22038.9	{16272., 24346.}	12.4084	0.0630981	newly 5 weeks spec	10541.	7310.14	{2605., 9145.}	30.6504	0.208078	newly 5 weeks cumul	46907.	37636.4	{22901., 43347.}	19.7639	0.109383	newly 55 weeks cumul	87719.	60629.1	{27272., 73353.}	30.8826	0.193672
43	newly 3 weeks spec	12045.	10466.5	{10255., 10677.}	13.1052	0.061059	newly 3 weeks cumul	24152.	21704.2	{21262., 22053.}	10.1351	0.0464648	newly 5 weeks spec	14237.	12267.7	{12184., 12487.}	13.8321	0.0650984	newly 5 weeks cumul	52694.	46386.2	{45799., 46945.}	11.9707	0.0554388	newly 60 weeks cumul	95113.	88670.3	{89146., 89513.}	6.77375	0.0313097
44	newly 3 weeks spec	16196.	15792.1	{13961., 16535.}	3.62979	0.0167178	newly 3 weeks cumul	32797.	32992.6	{31612., 34331.}	3.04253	0.0132922	newly 5 weeks spec	14170.	10619.8	{5510., 12062.}	25.0546	0.139226	newly 5 weeks cumul	64055.	58472.	{47434., 61362.}	8.71593	0.0413178	newly 60 weeks cumul	95957.	72848.9	{50913., 76280.}	24.0817	0.123784
45	newly 3 weeks spec	16795.	16585.7	{16130., 17080.}	2.1117	0.00927587	newly 3 weeks cumul	31747.	31783.9	{30517., 32960.}	2.41368	0.0104771	newly 5 weeks spec	14996.	14180.6	{13861., 14524.}	5.43738	0.0243685	newly 5 weeks cumul	65154.	63585.5	{62596., 64578.}	2.41635	0.0106567	newly 62 weeks cumul	96985.	89214.6	{87809., 90294.}	8.01199	0.0363208
46	newly 3 weeks spec	15111.	13619.6	{7789., 16375.}	14.4223	0.0838513	newly 3 weeks cumul	25292.	24801.7	{10732., 33437.}	25.2281	0.132023	newly 5 weeks spec	17991.	7167.93	{3971., 12066.}	60.1582	0.436035	newly 5 weeks cumul	63693.	44322.8	{38003., 47924.}	30.4119	0.159964	newly 64 weeks cumul	97957.	49291.6	{48191., 50379.}	49.6804	0.298334
47	newly 3 weeks spec	13356.	11851.7	{11346., 12350.}	11.263	0.0521544	newly 3 weeks cumul	27095.	25155.7	{23861., 26658.}	7.18664	0.0327336	newly 5 weeks spec	14547.	10458.8	{10197., 10752.}	28.1035	0.143388	newly 5 weeks cumul	57180.	48138.4	{46680., 49475.}	15.8125	0.0748663	newly 59 weeks cumul	92154.	66809.9	{66236., 67457.}	27.5019	0.139683
48	newly 3 weeks spec	14508.	13894.	{12950., 14546.}	5.04577	0.0227019	newly 3 weeks cumul	25267.	24946.4	{22917., 26510.}	4.06827	0.0179226	newly 5 weeks spec	17877.	15102.8	{14094., 15611.}	15.5182	0.0735587	newly 5 weeks cumul	62499.	57108.8	{54517., 59254.}	8.64086	0.0394692	newly 62 weeks cumul	95356.	85548.4	{77421., 90350.}	10.2852	0.0480264
49	newly 3 weeks spec	15110.	11301.	{10038., 12750.}	25.2086	0.12831	newly 3 weeks cumul	34829.	28608.	{26310., 30393.}	17.8617	0.0862625	newly 5 weeks spec	12499.	7659.88	{5451., 10925.}	38.7161	0.232416	newly 5 weeks cumul	62570.	46349.4	{40776., 53583.}	25.924	0.133489	newly 60 weeks cumul	88106.	61872.4	{48717., 83485.}	29.7751	0.166741
50	newly 3 weeks spec	13796.	12978.9	{11877., 13475.}	5.92694	0.02708	newly 3 weeks cumul	28249.	27569.1	{26108., 28470.}	2.94616	0.0131817	newly 5 weeks spec	14245.	11747.2	{8229., 12874.}	17.5345	0.0901762	newly 5 weeks cumul	58206.	53053.9	{45435., 55488.}	8.85142	0.0414714	newly 61 weeks cumul	94140.	79850.6	{56827., 86483.}	15.1789	0.0772172
